# Family medicine, ‘La Herencia’ and breast cancer; understanding the (dis)continuities of predictive genetics in Cuba

**DOI:** 10.1016/j.socscimed.2010.09.053

**Published:** 2011-06

**Authors:** Sahra Gibbon

**Affiliations:** Anthropology Department, University College London, UK

**Keywords:** Family medicine, Genetics, Inheritance, Breast cancer, Health beliefs, Cuba

## Abstract

Building on social science research examining the relationship between genetic knowledge, identity and the family this paper takes the cultural context of Cuba as a site for critical ethnographic engagement. The paper makes use of research working with a range of Cuban publics and genetic professionals as part of a collaborative research project exploring the social and cultural context of health beliefs about breast cancer. It illuminates the contrasting ways in which genomic knowledge linked to an increased risk of breast cancer is perceived, communicated, and acted upon. It is argued that the particular meaning and significance of genetic risk linked to breast cancer in this context must be examined in relation to long standing institutional practices relating to public health care provision. The focus on ‘the family’ in the provision of Cuban health provides a particularly viable foundation for the expansion of what is described as ‘community genetics’, including the collation of family history details for common complex diseases such as breast cancer. Nevertheless specific public perceptions of risk related to breast cancer and the difficulties of discussing a diagnosis of cancer openly in the family point to the very specific challenges for the translation and application of predictive interventions in Cuba. In summary the dynamic interrelationship between public health, perceptions of risk or health beliefs about the causes of the disease and attitudes towards cancer diagnosis within the family point to both continuities and discontinuities in the way that genomic interventions linked to breast cancer are unfolding as part of a dynamic yet still ostensibly socialist project of health care in Cuba.

## Highlights

► Intersections between genetic knowledge, the family and public health in Cuba. ► The meaning and significance of genetic risk linked to breast cancer. ► Community Genetics, family and the expansion of genetic medicine. ► Hereditary factors, gender and the significance of a ‘blow’ to the breast. ► ‘Continuities’ and ‘discontinuities’ between genetic medicine and public health.

## Introduction

The multi-layered dynamics between genetic knowledge or technology and family relations are now being explored in a variety of ways across the social sciences. Early work examining how common sense ideas of heredity (Davison, Frankel, & Davey-Smith, 1989) and kinship (Richards, 1996) inform notions of genetic inheritance have been extended to show the complex ways that family and kin relations are being ‘thrust into relief’ (Featherstone et al., 2006) and, in some cases, themselves transformed by genomic interventions. A few of these studies point to the way that novel genetic knowledge is concretising a ‘bio-genetic’ conceptualisation of the family that challenges diverse kin groupings, as well as the ideology of individual choice (Finkler, 2000). Others highlight the way genomic knowledge can be linked to novel forms of what have been described as ‘biosociality’ (Rabinow, 1996) that give new significance or meaning to ideas of personhood and social relations in the family and the wider community (see for instance Silverman, 2008).

Previous research undertaken in the UK, suggests that the particular arena of medicine characterised as ‘breast cancer genetics’ offers an important context for examining the relationship between identity, genetic medicine and the family. Following the identification of the two inherited susceptibility genes BRCA1 and BRCA2 in the mid 1990s the growth and emergence of this new area of clinical practice has provided a rich context for examining the relationships between genetics and kin or family relations, both inside and outside the clinic. This has revealed the often contradictory consequences of genetic knowledge for collective and individual identity. On the one hand something of a productive fit between the field of medicine characterised as BRCA genetics and the family has been identified. This has been linked to gendered notions of female nurturance (Gibbon, 2007a) or ideologies of the ‘traditional’ family as constituted by ideas of bio-genetic relatedness (Finkler, 2000). At the same time other research has also revealed a tension between a particular individual’s investment in what is perceived as preventative health and the relational consequences of predictive risk information for the family (Gibbon, 2007b; Hallowell, 1999). As the work of Konrad examining predictive genetic knowledge in the context of a different condition, Huntingdon’s Disease, has demonstrated, genetic knowledge can play out unevenly in the family. Such studies illustrate how interdependent social and personal relations between kin can be negotiated as ‘rights to know’ or ‘not to know’ about predictive risk information (Konrad, 2005, see also Featherstone et al., 2006).

Detailed case studies are now beginning to examine just how varied the consequences of genomic knowledge can be within the context of family or for community and social relations, depending on the disease condition and the cultural meanings that may be linked to it. While Weiner (2010) notes how genetic risk information may have little consequence for biosocial relations for those with Familial Hypercholesterolemia, Lock, 2008 points to the lack of novel biosocial organisation around the genomics of Alzheimer’s disease (2008). She points out how this possibility is itself over-determined in part by the difficult task of family care-giving for an incurable and often devastatingly debilitating condition. As the work of Rapp, 1999 demonstrates examining one very specific aspect of reproductive medicine - amniocentesis, there is still much to be done within Euro-American societies in understanding the way that genetic knowledges and technologies are informed by a variety of family contexts, class, gender, religion and ethnicity (see also Shaw, 2000; 2009). Yet comparative anthropological studies *outside* of Euro-American contexts are also now demonstrating how very different notions of personhood and meanings associated with kin, family, and/or citizenship have specific consequences for the meaning of and degree of engagement with genetic information. More broadly these studies point to the way differently situated moral values relating to spiritual, religious or community practices and institutional cultures, as well as state provision (or the lack) of health care may influence, facilitate or impede the application of novel biological knowledges and technologies (Gammeltoft, 2007; Gibbon & Novas 2008; Gibbon & Reynolds 2009; Inhorn, 2008; Kampriani, 2009; Sleebom-Faulkner, 2010).

This paper drawing on critical comparative ethnographic research in the specific cultural context of Cuba contributes to the task of illuminating the limits and varieties of ‘co-production’ (Jasanoff, 2004; Lock et al., 2000) in the relationship between genetic medicine and the family. With a public health system that has long aimed to provide comprehensive health care, based on the values of equitable and universal access, Cuba provides a unique arena for exploring the evolving relationship between genetic medicine and the family. Despite a lack of financial and technological resources to undertake widespread clinical predictive genetic testing for conditions such as breast cancer, there is an ongoing commitment in this context to mobilise what is described as ‘community genetics’ as a public health endeavour. In Cuba this increasingly includes the collection and analysis of family history data linked to common complex diseases such as breast cancer. This paper examines practices of ‘community genetics’ in Cuba, as this relates to an expanding interest in conditions such as breast cancer and the ongoing engagement in family medicine as part of state public health care provision. It demonstrates how Cuba provides an important comparative arena for exploring the continuities and differences in the relationship between genetic knowledge and technologies, personhood, kinship and the family.

## Research methods

The analysis in this paper is based on research that formed part of a collaborative project working with teams of Cuban genetic professionals in three different provinces in the east, centre and west of the country at various time periods between 2006 and 2008. The most intensive period of research was undertaken between September 2007 and March 2008. All regions where the research was undertaken are areas with their own particular pre and post revolutionary history of socio-economic development;, these are notably different to the Havana context (see Rosendhal, 1997). Data collected included ethnographic findings working alongside Cuban community genetic health practitioners, visiting families in their homes as part of the routine collection of family history information and also use of a semi-structured questionnaire. The questionnaire was completed by 250 Cuban women in 3 different provinces of the country. Topics covered by the questionnaire, which included both open and closed questions, focused on health beliefs concerning the perceived causes of and risk factors for breast cancer, including genetic and what were described as ‘non-genetic’ factors. The age range of participants completing the questionnaire was 16–80 and all were women. Of this group half had in the past, or in a few cases were currently being treated for breast cancer. Sampling and recruitment of research participants was the responsibility of the Cuban collaborators. Liaising closely with local polyclinics and family doctors they identified and invited participants to take part in the research. No systematic selection of participants was made on the basis of a family history of breast cancer, however approximately a quarter of research participants had family members affected by breast cancer or another type of cancer. Following the completion of informed consent procedures, the questionnaires were undertaken with participants mostly in their own home. Community genetic practitioners were present at all times and were responsible for writing in the research participants verbal response to the listed questions. The research was granted formal ethical approval by the PI’s host institution, University College London in 2006. The data generated by the research was both qualitative and quantitative and is being analysed in a variety of ways, using *SPSS* and *ATLAS ti* data analysis software. Data presented in this paper draws from both the ethnographic component of the project and selected aspects of questionnaire data. This includes responses to a range of open-ended questions asking participants about their perceptions and beliefs concerning the causes of breast cancer, what participants perceived as the most important risk factors for the disease and their personal (if relevant) or general experience of breast cancer in the family or community. These responses have been analysed based on a grounded theory approach drawing on a thematic analysis of re-occurring topics (Corbin & Strauss, 1990) using ATLAS ti software. While some other aspects of this research have been discussed elsewhere (Gibbon, 2009; Gibbon, Kampriani, & zur Nieden, 2010), other findings will be published as analysis of the large and diverse data sets generated by the research are completed.

The first half of this paper describes the context and emergence of what is described as ‘community genetics’ as part of a programme of public health in Cuba. It suggests that there is significant continuity between the comprehensive provision of public health care and the recent expansion of community genetics that relates directly to the long standing interest in and provision of what is described as ‘family medicine’. The second half of the paper draws more directly on the questionnaire data with Cuban women, focusing on the cultural meaning of breast cancer and health beliefs associated with the causes of the disease. This includes a perception that breast cancer is caused by factors that arise from outside and impact on the body and the significance (or not) of genetic or what is understood as hereditary risk. This part of the paper reflects on the challenges to the practice of community genetics given both health beliefs relating to the causes of the disease and also the climate of fear and silence within the family associated with discussing a cancer diagnosis. The data presented in the second half of the paper suggests that despite the institutional culture that has long been focused on the health of the family, the meeting points between an expanding area of community genetics linked to identifying and acting on increased genetic risk of breast cancer, may not be so easily aligned and are in fact characterised by a range of discontinuities, tensions and differences.

## Public health, family doctors and community genetics

The development of a comprehensive public health system in Cuba emerged out of the success and commitments of the revolution in the late 1950’s. Since that time Cuban health care has long stood as an important symbol of the success of the Cuban socialist revolutionary efforts (Brotherton, 2005; Feinsilver, 1993). This process of transformation included the creation of local primary health care services in the 1960’s and 70’s with locally based polyclinics set up across the country; including areas where health care resources had previously been scarce or non-existent. In conjunction with this tens of thousands of medical professionals were trained in the following decades with official government health statistics suggesting in 2001 that there were 31,000 doctors or nurses or one for every 175 people (MINSAP Cuban Ministry of Public Health, 2001). This enormous increase in medical professionals, combined with a focus on maternal health has been seen as directly responsible for the high profile successes of the Cuban public health care system. This is particularly with respect to reducing infant mortality and increasing life expectancy (Spiegel & Yassi, 2004). As a result despite the economic challenges of the ongoing US embargo and the collapse of the Soviet subsidies in the 1990s, the epidemiological profile of the country has been transformed from one with ‘diseases of poverty to diseases of development’, that now prominently include heart disease and cancer. According to the Cuban National Cancer Registry over 2000 cases of breast cancer are diagnosed annually in Cuba with a population of about 11 million inhabitants, making the incidence about 40 per 100,000 inhabitants (Alvarez, Garrote, Torres Babie, Guerra, & Jordan, 2003). Although there are regional variations within the country breast cancer is the most common malignancy affecting Cuban women with incidence of the disease comparable to a global rate and increasing every year (Galán et al., 2009).

Many commentators have suggested that understanding not just the symbolic significance of the ‘revolution’ in health care in Cuba over the last 50 years but also the logistics of its success, must be attributed to the system of so called ‘family medicine’ that began to emerge in the 1980s. The effort to provide more and better equipped family doctors who could attend to both the ‘physical and social well-being’ of the Cuban population was consolidated by the state in 1984 as part of the Family Physician and Nurse Programme (MEF) (Nayeri, 1995). This laid out the plans for the current organisation of primary health care with doctors working in local ‘consultorios’ in the communities in which they lived. By 1995 this system was established over the whole country integrating hospitals, local polyclinic services and community based doctors. This enabled Cuba to apply and, in the view of some, to a certain extent realise the principle of ‘health for all with a primary focus’ (Spiegel & Yassi, 2004: 97). Importantly prevention and not just treatment was a vital part of this medicine in the community programme, with health being seen somewhat holistically as a function of ‘biological, environmental and the social well-being’ (Nayeri, 1995: 324). This went far beyond clinical intervention to include ‘disease prevention, hygiene instruction, family planning and risk factor assessment’ (Jenkins, 2008: 13) see also Nayeri, 1995 and Brotherton, 2005.

The emergence of ‘Community Genetics’ in the last six years, as part of a national programme of intervention, emerges directly out the ‘holistic’ and ‘preventative’ focus on family medicine and a long standing, nearly 40 year old programme of infant and maternal health. This is in part reflected in the way that many professionals now working in the field of community genetics previously worked as family doctors in their local communities, before retraining to become specialists in genetics. It is also reflected in the infrastructure for this new health focus and the way that it is being consolidated and linked to the system of primary care set up through programmes such as the MEF, with networks of genetic centres and clinics. Employing a total of more than 1600 persons, across the country, many regional centres have their own dedicated genetic specialists, technicians and nurses (Teruel, 2009). Such centres, are often linked to polyclinics or local hospitals, but frequently also have their own designated buildings in residential areas with separate consulting rooms and laboratories.

The main day to day focus of work in these centres is newborn neonatal screening to monitor for rare chromosomal conditions and in helping to facilitate the national programmes of pre-natal screening for conditions such as sickle cell anaemia. By comparison, the work of genetic teams in relation to complex adult onset conditions such as breast cancer is focused on the collection and collation of registries of families affected by such conditions. In some centres the list of conditions for which family history was being collected included, at the time of the research, schizophrenia, Alzheimers, heart disease, diabetes and more behavioural type conditions such ‘alcohol addiction’. With in total over 43,000 families forming part of a national registry, this is potentially a powerful resource for future genetic research and medicine (Teruel, 2009). Yet due to the cost and lack of technological infrastructure, these centres are as yet unable to provide comprehensive clinical risk assessment or predictive information based on genetic testing for those with a family history of breast cancer. Newly established community genetic clinics are nevertheless engaged in the task of collecting family history data and identifying persons and families most at risk. It's important to note that such work was also propelled by the collaborative project that formed the basis of the research from which the data presented in this paper is derived. At the same time this project was for Cuban collaborators essentially an ‘exploratory’ study that would help assess and perhaps in the future expand the practices of community genetics in relation to breast cancer, it was also a means of enabling and facilitating the task of collating family history information.

Moving around rural and urban communities with different teams of medical geneticists in the three different provinces where the research for the project was undertaken it was clear that the genetic professionals were very much at the centre of their communities. Many had worked for years as family doctors living in or near the district in which they worked. Now as part of the program of Community Genetics, they could not walk down the street without encountering people they knew or more usually were stopped by people that recognized them. Frequent humorous comments were made by them about how their homes were like ‘consultorios’ every night, with neighbors, friends and acquaintances calling by to ask advice about health problems. On one occasion walking back to the community genetic clinic in the residential area of a small town in an eastern province, the doctor I was with was recognized and stopped by a mother and her teenage daughter. This was to discuss the fact that the daughter might be pregnant, and would therefore need an abortion. She had stopped the doctor to ask whom she should see at the local polyclinic about this. With a mixture of dismay and dry humour, about being so frequently stopped in the street and asked such queries, Celeste the doctor laughingly said that was community genetics in practice ‘es genetica communitaria en realidad!’. This social position at the heart of the communities where these health professionals lived was however particularly important in relation to the collation of family history details, as the experience of working with these health professionals illustrated.

It is true to say that the social context of health beliefs and practices in relation to breast cancer, the main focus of our collaboration, was of some interest to these health professionals. They were attuned to consider these aspects of health and well-being, primarily as a result of prior involvement in a broad based system of family medicine orientated towards a ‘holistic’ preventative approach. Nevertheless this involvement was for them also explicitly about the task of recording and registering family history or identifying high risk families. It was not perhaps surprising therefore that they quite often literally took charge of these moments of completing the questionnaire, sometimes busily drawing up mini clinical family trees or firing further questions about dates or details relating to the history of cancer in the family. Yet unlike the sometimes tense atmosphere that such questions could generate in the clinical contexts in the UK, it soon became obvious that these were routine and expected questions for both practitioners and patients in Cuba. The ease with which such information was exchanged with medical practitioners, was particularly evident when, as was frequently the case, other family members became part of the discussions in the hunt for details of the history of disease. This was illustrated one afternoon walking around a residential housing area with two members of the local genetic clinic, after completing the questionnaire with an elderly woman in a nearby block of flats. On encountering the nephew of the elderly research participant, whom the geneticist also knew, he was asked if he knew about a particular cousin’s medical history, as his elderly aunt had been unable to remember. He was not surprised to be stopped in the street while cycling back home from work by the geneticist whom he also knew and responded with good humour and no hesitation to the request to clarify the details that his older relative had roughly sketched out.

Asking and giving information about family history is part of the routine patient/practitioner dynamic in Cuba which the expanding field of ‘community genetics’ taps into and builds from. As the previous example demonstrates this was particularly evident in smaller communities where the relationship between genetic practitioners and the public was highly localised. Another illustration of this was the way genetic professionals would often become concerned that participants completing the questionnaires responded with what they perceived as the ‘correct answers’. Similarly when questions elicited blank or non-responses from participants, the geneticists would comment with open consternation, sometimes attempting to prompt participants and stating as one practitioner said, ‘ they do know the answers!’.

Cuban geneticists, in their attention to the family, ante-natal and new born care are like other doctors powerful and embodied symbols of the revolution. The high profile programme of ‘Internationalism’ involving the export of thousands of doctors to Africa, Asia and South America for periods of one to three years has been central to this symbolic association of doctors with the revolution both within and outside Cuba (Feinsilver, 1993). Importantly over the last few years the first ‘medical missions’ involving Cuban Community Genetic professionals to Venezuela have also taken place. This identification of genetic professionals with the ethics of the socialist state and ‘revolutionary values’, linked to ideals of equality of access and universal care of the population was reflected in the visual references in the genetic clinics themselves. Here hand made murals, public health messages would sit alongside Josi Marti poems and pictures of ‘Che’, Fidel Castro and Hugo Chavez. As the collaborative project linked to the research was undertaken in different provinces, a number of health professionals revealed their commitment in working in this way. An event recounted from field notes illustrates how these sentiments manifested themselves for one community genetic practitioner.

Mayra’s pride in showing me the newly built community genetics clinic, for which she is director, is evident. Unlike the generally old mainly very rundown buildings, the community genetics centre stands out as a gleaming newly painted building on a hill overlooking a provincial town in one of the eastern provinces of the country. Its location next to the maternity hospital in part symbolically reflects the way that Cuban Community Genetics builds on and extends the success of a widespread programme of maternal and infant health care. Inside the newly built centre the newness of the fixture and fittings is evident with work still going on to complete the centre. It is also one of the few buildings in the town with air conditioning. As we are walking around the as yet unused freshly painted conference room, which will be used for regional meetings of community genetic health professionals, conversation drifts into discussion of how Mayra got to be director of the regional genetics clinic here in this small town. She talks of being a family doctor in the Sierra Maestra in the difficult so called *Special Period* following the collapse of the Soviet subsidies in the late 1980’s and how this inspired her in her work. Since then she always wanted to be able to come back to the town where she had grown up and work. After doing her training in genetics she welcomed the opportunity to set up the community genetics service in her home town. Mayra’s dedication to the work is in fact noted by others who work in the centre - one nurse making some humorous yet nevertheless pointed remarks had earlier said in a semi ironic way how unlike the ‘other doctors’ who have left Cuba, Mayra is a ‘good communist’. Discussion with Mayra turns to talks of how she is going to put plants on the outside of the building to make the area more comfortable and welcoming for patients. She also mentions there is a sculpture that will be put up at the front of the building. She takes me to a small room where the sculpture which had been destined for this spot is currently being stored. It is a very classic representation of double stranded DNA. But she tells me that they aren’t going to use this one - she says it’s ‘feio’-ugly. The sculpture that will now be placed at the front of the building is something much more abstract and organic rather than obviously an object representing DNA or scientific knowledge. The choice of public sculpture at the entrance to the newly built community genetic initiative seems to symbolically reflect an effort to represent and position the work of medical genetics as part of the larger long standing project of community public health care. That is as a normalised aspect Cuban health care that directly builds on a long standing programme of public health, rather than something obviously novel or different.

Long standing investment and organisation of public health in Cuba has in fact placed locally and community orientated family medicine at the heart of an endeavour, from which community genetics extends and builds from. This situation would also seem to provide a certain degree of leverage for the growth and expansion of genetic medicine in Cuba, including that linked to BRCA genetics. As the ethnographic material outlined above suggests family doctors and community genetic professionals are a vital component of this endeavour, situated at the heart of locally organised system of health delivery that is centred on the family. At the same time their commitment to integrating community genetics into the Cuban project of public health, while not always as uncritically supportive or unaware of the resource challenges to this endeavour in Cuba (see Gibbon, 2009), is nonetheless central to the ongoing success and expansion of this field of medicine. The next section of this paper drawing on the questionnaire data with Cuban women and focusing on findings relating to participants’ health beliefs concerning inheritance and genetic risk examines the diverse and somewhat uneven ways in which an institutional culture of family medicine and community genetics informs the meaning of breast cancer genetics in Cuba. The local institutional culture of Cuban health care outlined in the first part of this paper, would seem to provide a fertile context for the continued expansion of community genetics as ‘family’ medicine. Nevertheless the findings from the questionnaire data related to health beliefs and the difficulties of talking about cancer in the family pose challenges to the implementation of predictive health interventions linked to breast cancer in the Cuban context.

### Understanding the meaning and morality of breast cancer risk; family history, ‘la herencia’ and ‘los golpes’

Analysis of aspects of the questionnaire data with Cuban women relating to open-ended questions concerning beliefs about the causes of breast cancer, risk factors for and the experience (if relevant) of the disease, points to the importance of a range of perceptions. In contrast to comparable research undertaken with clinical and non-clinical populations in the UK (see for instance Gibbon, 2007a) the meaning of ‘breast cancer risk’, as well as the morality normally associated with ‘health awareness’ and engagement in preventative health interventions, appeared to be somewhat differently articulated (see also Gibbon, 2009 and Gibbon et al., 2010 for further discussion of this contrast). Here two specific aspects of Cuban women’s health beliefs about the causes of breast cancer are examined. First the way that genetic and hereditary factors were understood and discussed. Second the way that a physical ‘blow’ or what was described as ‘um golpe’ is seen as the primary cause of the disease. I argue that this is illustrative of the way that risk of developing the disease is mostly seen as arising from and impacting on the body, rather than being generated within the body or as the outcome of individual actions (or inactions). Elsewhere I’ve explored this finding in relation to the findings of different sets of data, such as the significance of dietary factors and the meaning of ‘stress’ (Gibbon et al., 2010)

Given the relative unavailability of predictive genetic testing in Cuba, an absence of hype and hope-filled discussion of the ‘BRCA’ genes in the media, as well as the virtual absence of a strong culture of breast cancer activism (certainly outside of Havana), it was perhaps not surprising to find that very few of the women completing the questionnaire had heard of the ‘BRCA’ genes. Rather more surprising perhaps was the fact that many had no point of reference to the term ‘genes’ or ‘genetic’ factors. There would often be looks of bemusement and doubtful shaking of heads in response to open questions asking if people had heard of ‘los genes’ or ‘la genetica’. For those few persons who for whom the term ‘genes’ was meaningful, discussion centred on a vague notion of something perhaps being transmitted in the blood. This was how a number of persons expressed this;

‘Piensa que es algo que se transmite de una familia a otra en la sangre’

(I think that it’s something that is transmitted from one family to another in the blood).

‘Los genes son algo en la sangre que dan herencia y entonces para cancer tambien’

(The genes are something in the blood that is inherited and then the same for cancer also)

It was significant that when similar questions about genetic risk were re-phrased in terms of ‘hereditary factors’ (‘factores hereditarios’) there was a much more widespread positive recognition. That is to say there was much more likely to be discussion and understanding that ‘la herencia’ (inheritance) and ‘la salud o las enfermedades’ (the health or illness) of ‘los antecedentes’ or ‘antepasados’ (ancestors) might contribute to the risk of disease. This suggested that there was a particular cultural salience in this context surrounding hereditary risk, if not genes, genetic factors or more specifically the BRCA genes.

This was particularly evident in the way that direct questions relating to genes, as opposed to hereditary risk factors, could elicit both strongly negative and positive responses often from the *same* person. This was subtly illustrated in this respondent’s comments. Talking about where she had heard about the link between hereditary factors and breast cancer she said;

‘en documentales por la television, en conversaciones con personas se habla de que es un factor importante porque hay varias personas en las familias affectadas. De como es que los genes producen cancer no lo he escuchado’

(‘in television documentaries on the television, in conversations with people I’ve heard that its an important factor when there are different family members affected, but I haven’t heard anything about how genes produce cancer’)

Another woman was more cautious in her response;

‘pudiera ser una causa genetica, no ha escuchado en specifico sobre los genes en respecto de cancer de mama’

(‘you could say it was genetic but I haven’t heard specifically about genes for breast cancer’).

Other persons were more definitive with some incredulous in response to the suggestion that breast cancer was linked to hereditary or genetic factors ‘la herencia no esta vinculado con cancer de mama’ (inheritance isn’t linked to breast cancer’). In another instance two sisters who had both in fact had breast cancer talked much about their shared experiences of living through the treatment ‘somos gemelos en respecto de cancer de mama’ said one of them (we are ‘twins’ when it comes to breast cancer). However they both refused and strongly refuted when prompted that there was anything hereditary in the fact that they had both had breast cancer.

One woman quite explicitly in response to a query about genes linked to breast cancer made a clear distinction between unknown genetic factors and known hereditary risk;

‘No tengo conocimiento de esto [factores geneticas]pero piensa que pueda ser hereditario o sea que uno nazca con eso y se manifeste a cualquier edad’

(I don’t know anything about this[genetic factors] but I think that it could be hereditary or it’s that you are born with it and it can appear at any age’)

While ‘BRCA genes’ and genetic factors more generally had little point of reference for many research participants, hereditary risk factors were, as these examples suggest, more readily associated with the increased incidence of the breast cancer. That is while some felt that factors such as having a family history might be important in the development of breast cancer specifically, there was nothing like the kind of reading of breast cancer as a ‘genetic’ disease that accompanied the high profile announcements that heralded the hyped and hope-filled discovery and application of new knowledge of the BRCA genes in the mid and late 1990’s in the UK and other Euro-American societies (Gibbon, 2007a; Parathasarathy, 2007). It was also not insignificant that in responding to the questions conditions such as diabetes and asthma were in fact much more readily and easily linked to hereditary factors than breast cancer; both diseases which Cuban public health care directly attends to through the programme of family medicine. In summary the disjuncture between what were perceived as more meaningful hereditary factors and what might be described as unknown or unknowable genetic factors, suggested that while the former had a particular meaningful resonance in peoples’ lives, the latter did not.

This situation must in many ways be read in relation to the longstanding institutional culture and ideological values imparted through the Cuban project of public health care and the system of family medicine which has been in place in Cuba for the last 20 years. At the very least this ensures that attending to the history of disease in the family is commonplace. As one questionnaire respondent said in response to a query about why she believed hereditary factors were important in relation to breast cancer, ‘porque en las consultas siempre le pergunten si tienen antecedentes familiares con enfermedades’ (‘because in consultations they’re [doctors] always asking if you have relatives who are sick’). This was more succinctly and directly interpreted by one genetic professional who said ‘people are so used to questions about family history or thinking that it is important, because we are always asking them about it’.

It was certainly notable that in responding to direct queries about family history from genetic professionals many persons willingly exchanged such information. Moreover many had an impressive grasp of the details of their family medical history, remembering not only the dates a relative had died or been diagnosed but sometimes the details of the medical procedure they or a relative had undergone. Elsewhere I’ve argued that this impressive ability to be conversant in and recount the family medical history or be engaged with biomedical procedures, histories and scenarios, which often constituted a response to queries about the *experience* of breast cancer, reveals the extent to which *biologized* citizenship may be at stake in the Cuban arena (see Gibbon, 2009).

Nevertheless examining in more detail the particular way in which ‘embodied risk’ linked to breast cancer is understood by individual Cuban women provides another context for understanding the challenges and discontinuities in the translation of predictive health interventions linked to breast cancer in the Cuban context.

It is true to say that there was some discussion by a few research participants regarding the need to take care of one’s own personal health and that to neglect this, was perceived as being detrimental to well-being and might lead to disease. This was particularly evident for those few participants who lived or worked in or near tourist regions or who, because of family living abroad, had access to some limited but nevertheless welcome extra financial resources. Nevertheless a moral discourse about *individual* responsibility for health, which previous research in the UK had suggested was central to interest in and patient mobilisation around ‘BRCA genetics’ (see Gibbon, 2007a), was a far from obvious terrain of discussion for the majority of those who took part in the research.

For example so called ‘lifestyle risk factors’, although acknowledged by some as important to overall health, were not always seen as factors which individuals could easily personally or directly alter or effect. In general there was a feeling that the strongest risk or danger came from *outside* of and impacted *upon* the body. As I’ve argued elsewhere this often meant identifying risk factors which were not only outside of the control of the individual but sometimes outside of the control of the Cuban state. This might include pollution from international conflicts in Iraq or Afghanistan and environmental contaminants or ozone depletion (Gibbon, 2009) or a ‘deficit’ in dietary food intake (Gibbon et al., 2010). Here I explore this rendering of embodiment in relation to perception of risk related to breast cancer by examining the frequency and manner in which respondees explained the cause of the disease in terms of a ‘blow’ or in spanish‘ un golpe’.

It was notable that in response to questions about the causes or risk factors more than half of the total number of respondents thought that a ‘golpe’ was the primary or a secondary cause of or a factor in the development of cancer. While such descriptions were sometimes used interchangeably to reference both a physical blow that might have caused the disease or psychological trauma (sometimes also used to describe the experience of having breast cancer) it was the former meaning that was most evident in the response of participants.

For some it was a simply that a ‘blow’ was sufficient enough to ‘trigger’ a cancer; ‘un golpe puede desencadenar el cancer de mama’. For others a blow might be related to hereditary factors in more explicit ways. For instance this could be used to emphasise the causative function of the former ‘ no lo relaciona con la herencia, lo relaciona con el glope que recibio’(its not related to inheritance, its related to a blow that was received’). At other times this reasoning was inverted but in ways which still served to emphasise the importance of a ‘golpe’ in understanding the cause of breast cancer:

‘piensa que los mas importante es el factor hereditario, porque no todos en su familia han tenido golpes’ (she thinks that its related to hereditary factors, because not everyone in the family has had a blow[ to the breast].

For some it was a the fact that a physical blow might not be attended to in time which was perceived to be the problem. As one woman put it ‘los golpes que no se atendien bien pueden originar un coagulo y de ahi un cancer’ (the blows that aren’t looked after can develop a clot and then from there a cancer). This kind of statement reflected to some degree the importance of taking care of oneself, or ensuring that medical attention was sought. It also suggests that, at least for some, individual health awareness was not totally absent to this kind of reasoning.

It is perhaps notable that particular ideas of female gender were sometimes caught up with discussion of the way a ‘blow’ to the breast was perceived as a risk factor for breast cancer. There was frequent mention that the breast was a ‘zona delicada para las mujeres’ – a delicate and sensitive area of the body for women which was susceptible to inury. More telling of was the way inappropriate activity for women or what was described as ‘fuerza fisica’ or ‘phyiscal force’ was also implicated in the development of cancer. In the opinion of some this could be linked to the novel need for women to undertake physical work as a result of economic demands following the ‘Special Period’ or it might refer to womens’ recent involvement in traditionally male sporting activities, such as boxing or weight building. Such perceptions would seem to reflect, in part, anxieties about the changing role of women in Cuban society. Highly illustrative of such feelings was a comment from one participant who believed it had been the blows she had received from a rifle during the pre-revolutionary struggle which she had participated in the late 1950s that had caused her cancer; ‘el mas importante creo que es un golpe, yo recibi golpes cuando la clandestindad me golpearon con la culata de un fusil’(I think the most important is a blow I received when a I was hit by rifle but by a secret campaigner).

Others working in the US and Mexico have noted that ‘un golpe’ is not an uncommon explanation for breast cancer particularly among Hispanic (and also some non-Hispanic populations) (Finkler, 1991). Hunt points out in her work in Mexico, that a ‘golpe’ may perhaps function like the notion of ‘stress’ in the west, providing a ‘conceptual bridge’ and form of ‘moral reasoning’ between disorder in the body and society and therefore like the notion of stress provide ‘a flexible, versatile symbol, locating the source of the disorder within an individual life history’. But as Hunt also acknowledges, it importantly locates health risk in terms of an ‘an attack *from without*’ (my emphasis Hunt, 1998: 304). This suggests something slightly different from certain ‘western’ readings of stress that would normally centre on the culpability of the individual.

The frequency of recourse to explanations about the causes of breast cancer or risk of developing the disease as being linked to ‘a blow’ provides one illustration of the way that for many Cuban women the most important aetiological disease causing agents are those that impact *on* the person, body or self, rather than being generated from within or which arise as a result of an individual’s own actions. Such readings seem to reflect a perception of cancer as not a product of ‘the body at war with itself’, as is common in western readings of cancer, (Sontag, 1991; Stacey, 1997) but as being under attack or as result of the actions of ‘impersonal’ outside agents. This might partly be understood in relation to the context of economic shortage in Cuba, that commenced with what is commonly referred to as the Special Period following the collapse of the Soviet subsidies in the 1990s. Some suggest that this climate of shortage has helped to foster a culture of capitalism in necessary practices of barter and exchange (Brotherton, 2005, 2008). The qualitative data presented here relating to health beliefs about breast cancer, among participants outside of the metropole of Havana, suggests this climate also informs perceptions of embodied ‘future’ risk in ways that do not necessarily provide a viable context for an expanding field of predictive genetics.

A recent prominent discourse within social science which has emerged partly in response to and as a way of understanding the meaning and significance of identity in the context of genomics, has suggested and implied that there is strong ideological fit between the emergence of genetic knowledge and the expansion of what has been described as ‘self actualising’ personhood. In other words the idea that there is a moral obligation to take responsibility for one’s health is something of a pre-requisite for, as well as a consequence of novel biomedical technologies and genomic medicine (see for instance Rose & Novas, 2005). That is a particular form of ‘biological citizenship’ linked to a burgeoning culture of breast cancer activism which mobilises a preventative health ideology in its emphasis on individual vigilance and awareness, seems to have been an important aspect of the growth and expansion of breast cancer genetics in the UK and US (Gibbon, 2007a,b; Parathasarathy, 2007). There is also evidence that an emphasis on female health awareness is a more complex but nevertheless important feature of the way this field of medicine has expanded elsewhere, as Kampriani (2009) demonstrates in her work on Greece. In Cuba such public or individualised health activism, with respect to breast cancer, seems somewhat absent and differently configured. That is for the most part it not only that the actions of individuals seem not to be perceived as the primary cause of diseases such as cancer but that the most dangerous agents are located outside the body. This does not mean that a notion of individual responsibility is totally absent in Cuban public health discourse, which is itself dynamically responding to a changing political context and climate of health provision (see Brotherton, 2005). There were instances, as mentioned previously, in undertaking the questionnaires in certain parts of the country closer to the metropole of Havana or tourist regions in the country where evidence of a discourse of health awareness informed research participants responses to questions about risk (see Gibbon et al., 2010). Despite an awareness of and interest in ‘hereditary factors’ the critical differences in the way that embodied risk is configured by Cuban women who took part in the questionnaire study does suggest that this is a context in which one of the apparent requirements for the expansion of predictive genetic medicine are less readily and immediately visible.

The final section of this paper further illuminates this aspect by pointing to one of the difficulties that confronted genetic practitioners as they moved in their local communities collecting family history information about feared and still stigmatised diseases such as cancer.

### From community genetics to predictive medicine. The challenge of talking about ‘cancer’ in the family

While the long standing institutional culture of family medicine would seem to provide a logical starting point for the expansion of community genetics in Cuba to encompass predictive interventions, the reading of ‘breast cancer risk’ by Cuban research participants would suggest that this transition is not so easily achieved. Ethnographic research working with health professionals, highlighted an issue that became particularly evident in the course of undertaking the questionnaires with families in their homes; that is the challenge of openly discussing the diagnosis of ‘cancer’ in the family. Given the easy exchange of family medical history information that the practice and culture of community genetics and family medicine in Cuba would seem to facilitate, this difficulty seems somewhat contradictory. It reflects the dread and fear in Cuba that is associated with the modern disease of cancer and an ongoing culture of paternalism that affects public health practices.

The ability of health practitioners to access, and willingness on the part of patients and their families to provide information about family health history and other diseases is an essential aspect of the development of predictive genetic interventions. Even in Euro-American contexts, where the ability to carry out genetic testing is more widely available, accessing family history information is still of prime importance in risk assessment procedures, in decisions about whether to offer testing and in helping to establish the meaning of mutation and predictive testing (Guttmacher et al., 2004). The absolute need for collective family engagement in predictive genetic medicine is perhaps most readily evident when the requirement to share information and pass on risk information breaks down in the family, impeding and sometimes preventing the pursuit of a genetic risk diagnosis for different members of the family (Gibbon, 2007b; Hallowell, 1999; Konrad, 2005). Despite the fact that the institutional culture of family medicine in Cuba seems to facilitate the collection of family history information and potentially at least the future expansion of this arena of medical intervention, the inability often to discuss and share a diagnosis of cancer between a patient and their family poses a significant practical and ethical challenge for community genetics in Cuba. That is while a family history of medical procedures and hospital interventions may have been widely known and remembered by patients and their families, the specific diagnosis of cancer was sometimes couched in more metaphorical terms or more problematically simply absent from individual and collective conversations.

This was illustrated on a number of occasions when working with Cuban health professional. On such occasions on arriving at the home of a potential research participant we would be met by an anxious relative of the chosen research participant. While they were often happy for the research questionnaire to be given to the person in question, they were concerned about their relative (normally in these situations a mother, grandmother or aunt) being told that they currently had or had in the past been diagnosed with cancer or ‘el cangrejo’ – the crab, as sometimes the disease was metaphorically described. On some occasions this difficulty led to a decision to not undertake a questionnaire. In other moments when it was less clear if a relative had been told or not if they had or had had cancer open-ended questions were re-phrased by community genetic practitioners to avoid the use of the word ‘cancer’. In these situations, ‘problemas con las mamas’ (breast problems), ‘nodulos’ (breast lumps) and other euphemistic terminology would be used in circuitous ways by both practitioners, relatives and sometimes also research participants themselves in responding to questions. As recent studies in Cuba have suggested the tradition of non-disclosure of cancer diagnosis to the patient (Roll, Simms, & Harding, 2009), reflects a degree of long standing paternalism in the public health system. This, coupled with a dynamic of care within the family which assumes that not telling a relative their diagnosis of cancer is the best course of action, constitutes a series of problematic challenges for genetic practitioners in undertaking many aspects of their work. This includes the requirements for meeting ethical guidelines relating to individual informed consent (see Gibbon, 2009). An inability to provide widespread predictive testing ensures that in part the problems of sharing future genetic risk information in the family do not constitute a significant challenge as yet in the Cuba. Nevertheless the problems of discussing a diagnosis of cancer within and between the patient and their family reflect a significant point of rupture and discontinuity in the translation of predictive medicine in the Cuban arena.

## Conclusion

This paper, drawing on fieldwork with a cohort of Cuban women and working with Cuban genetic health professionals has explored some of the uneven and disjunctured dynamics that characterise the translation of genetic medicine linked to breast cancer in a very specific national/cultural arena. It has been argued that the continuities and discontinuities that characterise this process must be situated in relation to a nexus of social practices and cultural discourses that both enable and challenge this endeavour in different ways. This includes the long standing institutional culture of Cuban public health care provision, research participants beliefs or perceptions about risk relating to breast cancer and the challenge of openly discussing a cancer diagnosis between patients or health practitioners in the context of the family.

In one sense the Cuban case seems to point to the presence of particular continuities in the practice and provision of public health and novel interventions related to assessing genetic risk in common complex conditions such as breast cancer. Ethnographic evidence suggests that long standing locally organised aspects of the health care system, focused on family medicine, has helped to give meaning and significance to the idea of family history as a risk factor for disease whilst also furthered the emerging medical arena of community genetics. That is the work of collating family history and managing the delicate inter familial relations that are part of genetic interventions, are central to a tacit practice of care-giving within the community. Here doctors are situated as paternal guardians and gatekeepers of this and other health information. Although without technological or financial resources to undertake widespread predictive testing, being able to match genealogical data to clinical records confers a degree of scope and flexibility to an emerging and changing practice of Cuban medical genetics.

The second half of this paper highlights how alignments between the focus on family medicine and moves to incorporate predictive health interventions in Cuba are in fact overlaid by difficulties and tensions that act as impediments to the easy translation of ‘BRCA’ genetics in this context. Three such aspects of these dynamics have been explored in this paper. Drawing on qualitative data examining health beliefs in relation to breast cancer the findings presented here suggest that while family history may be perceived to constitute a risk factor for many diseases (not just breast cancer), ‘genetic risk’ has little meaningful resonance for many research participants. At the same time particular perceptions of risk are informed less by a discourse of individual moral responsibility than by an understanding of risk and danger that is more likely to be located in cancer causing agenets that exist *outside* or operate *on* the individual. This paper has drawn on illustrative examples relating to the perceived causative effect of physical blows to the breast and gendered ideas about excessive and what is understood as ‘unnatural’ physical activity undertaken by women. While further research and analysis of the large and diverse data set will further illuminate this finding, the data presented here highlights the need to examine the varieties of biosocial identities that are at stake in the translation of genomic technologies across a diverse national and transnational global terrain (Gibbon et al., 2010). The final section of the paper points to a further difficulty linked to the effort to incorporate predictive medicine, that somewhat conflicts and would appear to be at odds with the institutional culture of family medicine in Cuba. Here the silences and use of metaphorical language that can characterise medical and family discourse relating to a diagnosis of cancer constitutes a significant ethical and logistical challenge to the translation of predictive interventions linked to increased risk of disease such as breast cancer.

In summary the very different configuration of factors that provide a context for the emergence of breast cancer genetics in Cuba provides a powerful illustration of the need for broader comparative perspectives in examining the relationship between genetic interventions and the family. The data presented here demonstrates the importance of examining the way that culturally and historically specific variables must be accounted for, as the work of translating predictive medicine is undertaken across comparatively different national arenas. This includes understanding the continuities, disjunctures and differences at stake in the dynamic relationship between genetic medicine, identity and the family.
